# Optimal but unequitable prophylactic distribution of vaccine

**DOI:** 10.1016/j.epidem.2012.03.001

**Published:** 2012-06

**Authors:** Matt J. Keeling, Andrew Shattock

**Affiliations:** Mathematics Institute & School of Life Sciences, University of Warwick, Coventry, CV4 7AL, United Kingdom

**Keywords:** Final epidemic size, Optimal control, Spatial metapopulation, Vaccination

## Abstract

The final epidemic size (*R*_∞_) remains one of the fundamental outcomes of an epidemic, and measures the total number of individuals infected during a “free-fall” epidemic when no additional control action is taken. As such, it provides an idealised measure for optimising control policies before an epidemic arises. Although the generality of formulae for calculating the final epidemic size have been discussed previously, we offer an alternative probabilistic argument and then use this formula to consider the optimal deployment of vaccine in spatially segregated populations that minimises the total number of cases. We show that for a limited stockpile of vaccine, the optimal policy is often to immunise one population to the exclusion of others. However, as greater realism is included, this extreme and arguably unethical policy, is replaced by an optimal strategy where vaccine supply is more evenly spatially distributed.

## Highlights

► Develops an efficient framework to determine epidemic sizes in structured populations. ► Optimal allocation of vaccine is highly heterogeneous. ► For low vaccine supplies may be optimal to focus on a single population. ► Greater heterogeneity within populations leads to more equitable allocations.

## Introduction

One of the great advantages of epidemiological models is their ability to discriminate between a range of plausible control options. For veterinary infections (such as foot-and-mouth disease (Tildesley et al., 2006, 2009), bovine-viral-diarrhea (Santarossa et al., 2005) or Johne's disease (Groenendaal et al., 2002)), where the main aim is to protect the majority no matter what the control, determining optimal control strategies is relatively straight-forward although potentially computationally intensive. In contrast, with human diseases control options are generally far more limited and hence research often focusses on the best means of deploying vaccine. Four main approaches dominate: the desire to minimise the basic reproductive ratio (*R*_0_) for a given supply of vaccine administered prophylactically (Anderson and May, 1982, 1984; May and Anderson, 1984; Hadeler and Mueller, 2007; Tanner et al., 2008); the related issue of minimising the prevalence of an endemic infection by on-going vaccination (McLean and Anderson, 1988; Klepac et al., 2011), the highly complex issue of distributing vaccine during an epidemic so as to minimise total cases or costs (Clancy and Green, 2007; Gaff and Schaefer, 2009; Salathe and Jones, 2010; Brown and White, 2011; Buonomo, 2011; Knipl and Roest, 2011; Shim, 2011); and the situation considered here where prophylactic vaccination is targetted to minimise the total expected epidemic size (Longini et al., 1978; Dushoff et al., 2007). In general three main techniques dominate: the use of simulation or numerical integration to compare strategies (McLean and Anderson, 1988; Bansal et al., 2006; Hall et al., 2007; Medlock and Galvani, 2009; Perisic and Bauch, 2009; Keeling and White, 2011; Salathe and Jones, 2010; Tuite et al., 2010) which is often the only option with complex transmission models; the use of game theoretic approaches to compare individual-level decisions with nationally optimal policy (Bauch et al., 2003; Galvani et al., 2007; Shim et al., 2009); and more recently the use of methods from control theory to minimise some associated cost function (often taken to be proportional to the integral of the prevalence and the square of the vaccinate rate) (Becker and Starczak, 1997; Clancy and Green, 2007; Gaff and Schaefer, 2009; Brown and White, 2011).

Here we adopt a far simpler approach and goal, to determine the optimal prophylactic distribution of a limited stockpile of vaccine between various populations that reduces the overall magnitude of a subsequent epidemic. The advantage of considering prophylactic vaccination (as opposed to vaccination during an epidemic) is that the final epidemic size can be calculated with relative ease even in a heterogeneous population – and more-over the results are independent of many of the precise assumptions concerning transmission. Here, we first motivate and introduce the general final epidemic size calculation; before using this quantity to examine the optimal vaccination policy in spatially segregated populations.

## Generality of the final epidemic size

The final epidemic size, defined as the total number of cases generated during an epidemic, was first formulated by Kermack and McKendrick (1927). In determining the final epidemic size, the usual calculation is to follow the methodology of Kermack and McKendrick and consider the final proportion (or number) of recovered individuals using the standard SIR model. However, far greater generality can be achieved if we utilise a probabilistic formulation. Consider a single individual that is susceptible at the start of the epidemic; given that infection is a Poisson process, the probability that the individual is still susceptible at the end of the epidemic is:

(1) $$\mathbb{P}(\mathrm{Susceptible})=\exp\left(-\Lambda \right)$$

(Barbour and Mollison, 1990) where Λ is the total force of infection experienced by an individual over the course of the entire epidemic (that is the integral over all time of the risk of infection). In a closed homogeneous population this total force of infection is:

$$\Lambda =\frac{R_{0}}{N}\times \mathrm{Total}\;\mathrm{number}\;\mathrm{of}\;\mathrm{cases}=\frac{R_{0}}{N}Z_{\infty }$$

where *N* is the total population size, and *R*_0_ (the basic reproductive ratio) is the expected number of secondary cases produced by an infected individual when the entire population is susceptible, and *Z*_∞_ is the total number of cases during the epidemic. This quantity Λ may be considered as the integral of the force of infection (which itself is a rate) over the entire epidemic; hence Λ is a dimension-less quantity that informs about the total risk of infection over the epidemic. Therefore, if we assume that the entire population is initially susceptible, and set the final epidemic size (*R*_∞_) to be the proportion of the population that get infected (i.e. *Z*_∞_ = *NR*_∞_) we arrive at the well known formula of Kermack and McKendrick (1927):

(2) $$R_{\infty }=1-S_{\infty }=1-\exp(-R_{0}R_{\infty })$$

which holds for all distributions of latent and infectious periods not just the standard exponential decay of classical ODE models. It should be stressed that this equation only holds for a large population size, where invasion can be guaranteed and stochastic variability in the final epidemic size is negligible.

We note that Eq. (2) can be solved using the simple recursive formula:

$$R_{\left(x+1\right)}=1-\exp(-R_{0}R_{\left(x\right)})$$

where we choose *R*_(1)_ = 1 for simplicity. This iterative scheme rapidly converges to the non-trivial solution for all positive values of *R*_0_ and for all non-zero initial conditions.

Given that Eq. (1) holds for all susceptible individuals, it is trivial to extend the classic final size equation to any structured population, whether this structure reflects age, social or spatial heterogeneties. In particular, suppose that we can subdivide the population into disjoint classes (denoted by super-scripts); then the probability that an individual in class *i* remains susceptible is given by:

$$\mathbb{P}({\mathrm{Susceptible}}^{i})=\exp(-\Lambda^{i})=\exp\left(-\sum_{j}\frac{R_{0}^{\mathrm{ij}}}{N^{i}}Z_{\infty }^{j}\right)$$

where $R_{0}^{\mathrm{ij}}$ is the matrix equivalent of the standard scalar *R*_0_, and measures the expected number of secondary cases produced in class *i* by a single infected individual in class *j*, assuming that all members of the *i* class are initially susceptible. *N*^*i*^ is the number of individuals in class *i*, such that ∑_*i*_*N*^*i*^ is the total populations size. Then dealing with numbers (rather than proportions) and assuming that initially $S_{0}^{i}$ (≤*N*^*i*^) individuals in class *i* are susceptible, we have:

(3) $$Z_{\infty }^{i}=S_{0}^{i}-S_{\infty }^{i}=S_{0}^{i}\left[1-\exp\left(-\sum_{j}\frac{R_{0}^{\mathrm{ij}}}{N^{i}}Z_{\infty }^{j}\right)\right]$$

In fact this relatively simple equation was first used by Longini et al. (1978) but remarkably has received little scientific interest since (although see Ma and Earn (2006), Dushoff et al. (2007) for exceptions).

We can attempt to solve the final size Eq. (3) using simple recursion:

$$Z_{t+1}^{i}=S_{0}^{i}\left[1-\exp\left(-\sum_{j}\frac{R_{0}^{\mathrm{ij}}}{N^{i}}Z_{t}^{j}\right)\right]$$

with initial conditions, $Z_{1}^{i}=S_{0}^{i}$. Whilst this iterative method is not guaranteed to converge for all initial conditions, when setting $Z_{1}^{i}=S_{0}^{i}>Z_{\infty }^{i}$ monotonic convergence to the solution is guaranteed. Numerical tests indicate that convergence is relatively quick unless the basic reproductive ratio is close to one in which case convergence may take up to one thousand iterations. Alternatively, simple search algorithms can be utilised that find the solution by minimising the difference between $Z_{t}^{i}$ and $Z_{t+1}^{i}$. Both methodologies provide a more computationally efficient method than direct simulation of the full dynamic equations, and allow a much more simple means of determining the error in the calculation of the final epidemic size. In addition, given that the final-size formulae is derived on probabilistic grounds, this implies that our results are robust to changes in transmission patterns; hence the results are invariant under different assumptions about the infectious periods (e.g. assuming fixed recovery rates or more complex distributions of infectious period times) or including more complex natural histories such as latent periods.

We now utilise this result to consider the optimal deployment of a fixed vaccine stock against a novel epidemic in a spatially structured environment.

## Non-interacting spatial communities

We begin by initially considering non-interacting spatial communities (hence setting $R_{0}^{\mathrm{ij}}=0$ whenever *i* ≠ *j*), such that the final epidemic size can be calculated independently for each using Eq. (2). We wish to consider the final epidemic size after a proportion of each community has been vaccinated prophylactically, and to determine the optimal deployment of a fixed amount of vaccine (*V*_*T*_) that minimises the total epidemic size. Mathematically, this can be expressed as:

(4) $$\begin{array}{c} \mathrm{minimise}\sum_{i}Z_{\infty }^{i}\;\mathrm{where}\;Z_{\infty }^{i}=(N^{i}-V^{i})\left[1-\exp\left(-\frac{R_{0}^{i}}{N^{i}}Z_{\infty }^{i}\right)\right]\\ \mathrm{such}\;\mathrm{that}\sum_{i}V^{i}=V_{T}\;\mathrm{and}\;V^{i}\geq 0 \end{array}$$

**Fig. 1:**
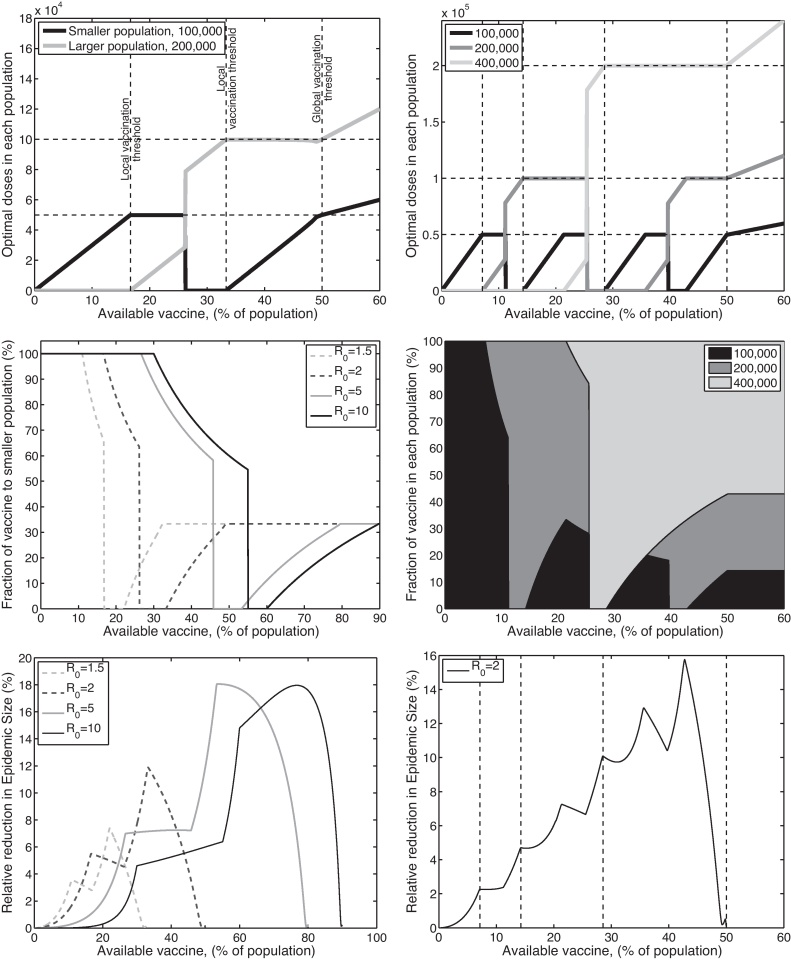
Examples of optimal control in isolated populations with homogeneous internal dynamics. The left-hand column has two populations of sizes 100,000 and 200,000, respectively; whilst the right-hand column has three populations of sizes 100,000, 200,000 and 400,000. Due to the simplicity of the two-population model (left-hand column) several values of *R*_0_ are considered (*R*_0_ = 1.5, 2, 5, 10), whereas for the three-population model we focus on *R*_0_ = 2. In the top row (graphs (a) and (b)), we show the optimal distribution of vaccine doses between the different populations as the total amount of vaccine available varies (in both graphs *R*_0_ = 2); the dashed horizontal and vertical lines correspond to the amount of vaccine required to bring each population to herd immunity. In the middle row (graphs (c) and (d)), we show the proportion of the available vaccine in each population at the optimum; in graph (c) we focus on the proportion in the smaller population and show the curves for different *R*_0_ values, in graph (d) the proportions in the three populations are shaded (for *R*_0_ = 2). Finally, in the bottom row (graphs (e) and (f)) we consider the relative reduction in epidemic size that can be achieved through the optimal deployment of vaccination compared to a homogeneous distribution ($(R_{\infty }^{\mathrm{homogeneous}}-R_{\infty }^{\mathrm{optimal}})/R_{\infty }^{\mathrm{homogeneous}}$).

We begin by considering the simplest case of two populations of different sizes, say *N*^1^ = 10^5^ and *N*^2^ = 2 × 10^5^; the optimal deployment of prophylactic vaccination is shown in Fig. 1(a and c). A striking pattern is observed, when a only small amount of vaccine is available, it is optimal to vaccinate just the smaller population. Mathematically, we can attribute this to the non-linear relationship between the proportion of individuals infected in an epidemic (*R*_∞_) and the proportion of the population that are susceptible (*N* − *V*). Biologically, the intuitive explanation is that one dose of vaccine in a small population has a proportionally large effect, and takes that population closer to the critical vaccination threshold, than one dose in a larger population. Therefore, we observe that for small amounts of vaccine the optimal solution (in terms of minimising the total final epidemic size) is to concentrate all of this vaccine into the smallest population; this pattern continues until we reach the critical vaccination threshold of the small population (*V*_*T*_ = *N*^1^(1 − 1/*R*_0_)) at which point it becomes unnecessary to deploy any more vaccine into the smallest population and our attention switches to the other. However, at some point (and generally before we reach the critical threshold for the other population, *V*_*T*_ = *N*^2^(1 − 1/*R*_0_)) it becomes beneficial to concentrate all the vaccine in the larger population and to ignore the smaller hence the optimal strategy suddenly switches. The position of this switch does not have an obvious analytical or biological value. However, intuitively it occurs because close to the critical threshold for population 2, the greater benefit comes from being close to this eradication threshold and hence protecting the majority of a larger population at the expense of the smaller.

Similar patterns can be seen for a range of populations and sizes, Fig. 1(b and d) shows the optimal prophylactic vaccination deployment for three populations (*N*^1^ = 10^5^, *N*^2^ = 2 × 10^5^, *N*^3^ = 4 × 10^5^). From this we begin to see a general set of rules: for a fixed supply of vaccine we should first vaccinate the largest population (or combination of populations) in which we can achieve the critical vaccination threshold, with the remaining vaccine being deployed into the smallest remaining population. This pattern of all-or-nothing vaccination goes against our basic intuition and desire to give an equal coverage to all populations, but can be seen to have significant savings in terms of reducing the number of individuals infected (Fig. 1(d and e)). It should be noted that although these graphs (Fig. 1(d and e)) are not monotonic, as the difference between optimal and random vaccination in non-monotonic, the total number of cases prevented by vaccination does decrease monotonically with increasing vaccine availability.

## Interacting communities

In reality, no communities are completely isolated and so our independence assumption in Eq. (4) is a simplifying approximation. However, the methodology developed in Eq. (3) allows us to extend these concepts to populations that interact. In particular we look at two examples which extend the results above: a set of coupled spatial communities so that the force of infection within each patch is related to both the local and global prevalence of infection; and a set of uncoupled spatial communities, where the internal populations can be risk-structured.

**Fig. 2:**
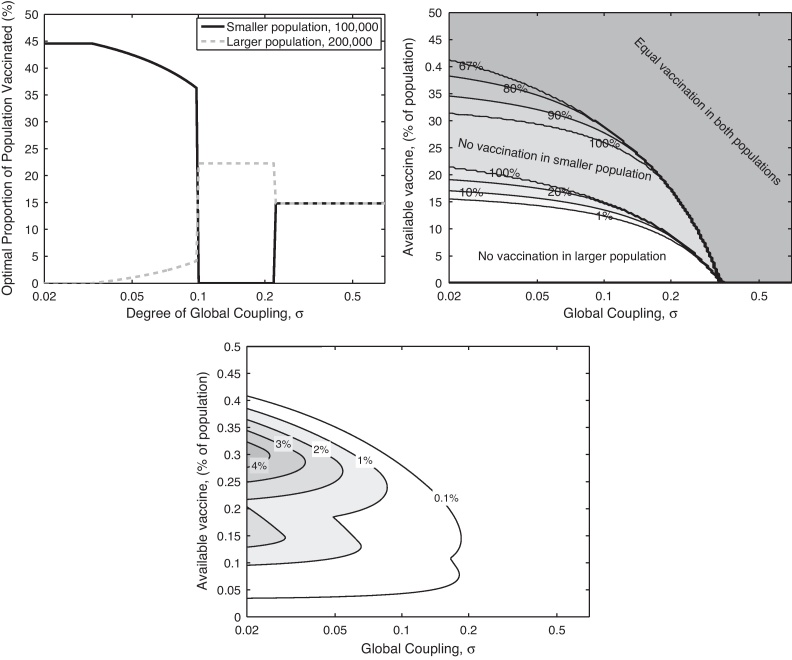
Effects of including epidemiological interaction (coupling) between populations. Graph (a) shows the optimal distribution of vaccine between two populations as the degree of coupling between them varies; note the *y*-axis now show percentage of each population vaccinated to more clearly illustrate when the optimal distribution is homogeneous (*R*_0_ = 2, population sizes of 100,000 and 200,000, and sufficient stockpile to vaccinate 15% of the population). Graph (b) shows the optimal proportion of vaccine distributed to the larger population as both the level of coupling (*x*-axis) and the stockpile of vaccine (*y*-axis) vary. For the optimal solution (shown in graph (b)) graph (c) shows the relative reduction in epidemic size compared to homogeneously vaccinating the populations.

For the coupled spatial communities, we set the degree of within and between transmission as:

$$R_{0}^{\mathrm{ij}}=\left\{\begin{array}{cc} \frac{R_{0}\sigma }{n} & \mathrm{if}\;i\neq j\\ R_{0}\left(\frac{1-(n-1)\sigma }{n}\right) & \mathrm{if}\;i=j \end{array}\right)$$

where *n* is the number of spatial communities (Keeling et al., 2004). This corresponds closely to the ideal of strong local transmission, with equal weaker global transmission to other communities. The balance between the local and global transmission is controlled by *σ* (*σ* = 0 gives purely local transmission, whilst *σ* = 1 means that all individuals experience the same force of infection irrespective of location); the precise formulation ensures that the expected number of secondary cases produced by an infected is independent of *σ*. Fig. 2(a and b) shows how the mixing between two communities can change the optimal strategy. For the parameters chosen in Fig. 2(a), when the communities act independently (there is no transmission between them and *σ* = 0) then it is optimal to exclusively target vaccination towards the smaller population, however as the level of interaction increases the optimal strategy rapidly switches to targetting the larger population before switching again to an even distribution of vaccine. Associated with shift towards a more even distribution is a natural weakening of the impact of targetting this prophylactic vaccination (Fig. 2(c)).

**Fig. 3:**
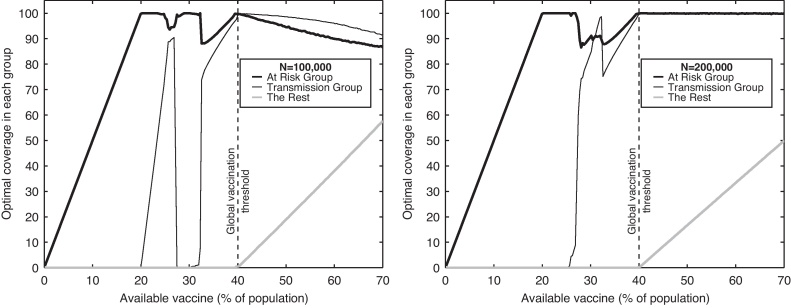
Optimal deployment of vaccination in two (isolated) structured populations. Three groups are considered within each population: at-risk individuals who suffer badly from infection (20%, thick black line), a group of high-transmitters who readily spread and catch the infection (20%, thin black line) and the remainder of the population (60%, thick grey line). Graph (a) and (b) show the percentage of each group in each population that should be vaccinated to minimise the total expected adverse effects from the epidemic. The within-population transmission structure is such that the basic reproductive ratio is 2, individuals in the high-transmission group are four times more likely to infected other members of this group compared to all other transmission rates which are equal. The high-risk group is considered to be five times more likely to suffer adverse effects from infection.

In the risk-structured populations (Fig. 3), we again have two communities (with population sizes of 100,000 and 200,000) but partition both communities into three further groups: at-risk, high-transmitters and the remainder – echoing the model framework in Keeling and White (2011). Here, at-risk individuals comprise 20% of the population and have greater risk of adverse health outcomes if infected; high-transmitters are a further 20% of the population, and can be thought of as school-age children who are known to be responsible for significant amounts of transmission. The three risk classes and two communities leads to a model with six populations, of sizes 20,000, 20,000 and 60,000 for community one, and 40,000, 40,000 and 120,000 for community two. The cost of infection in the at-risk group is assumed to be five times larger than the cost for other groups, capturing the greater impact of infection in this group, whilst the additional transmission within the group of high-transmitters is assumed to be to within this group, mimicking the observed strong within-school transmission patterns. To make the system more manageable we again revert to the simplifying assumption that the two communities do not interact. In Keeling and White (2011) vaccination within this type of model was examined for a single population through numerically solving the associated ODEs; here we take the simplifying approach of using Eq. (3) which greatly enhances the computational efficiency.

When such additional structure is added to the model, the optimal prophylactic vaccination policy again becomes more homogeneous (Fig. 3); although with six risk groups finding the global optimal solution becomes more problematic. For a small vaccine supply (less than 20% of the total population size), the available vaccine is shared equally between the at-risk groups in the two populations, echoing the single-population finding of other studies (Medlock and Galvani, 2009; Keeling and White, 2011). However, if the supply of vaccine is larger it is optimal to target the high-transmitters in the smaller population only, then the high-transmitters in the larger population only, then both of these groups together – echoing the earlier results in Fig. 1. When the supply of vaccine reaches 40%, it is possible to control the infection (set *R*_0_ = 1) by immunising all at-risk and high-transmission groups. For supplies above 40% it becomes optimal to begin vaccinating the remainder of the population, biased towards the smaller population at the expense of at-risk and high-transmission groups in this population.

The findings in Figs. 2 and 3 are typical for a range of parameters. Obviously as *σ* → 0 we regain the non-interacting results of Fig. 1, whereas as *σ* → 1 the population acts as homogeneously mixed and the question of where to vaccinate become superfluous. The patterns observed in Fig. 3 are insensitive to small changes in parameters; but if either the cost of infection in the at-risk group becomes relatively low, or the additional transmission within the high-transmission group becomes very high, it may become beneficial to initially target the high-transmission group (Dushoff et al., 2007).

## Discussion

In planning for future pandemics, such as a novel outbreak of (pandemic) influenza, one important public-health decision is how to distribute limited stockpiles of available pre-pandemic vaccine (Ferguson et al., 2006; Medlock and Galvani, 2009; Lee et al., 2011; Knipl and Roest, 2011). Pre-pandemic H5N1 vaccine is now available and stockpiled by many countries; the UK, Japan and the USA plan to stockpile, 3.3 million, 10 million and 40 million doses, respectively. The hope is that governments and public-health agencies will have sufficient advanced warning of an impending outbreak, that there will be time to administer the vaccine stockpiles and for the vaccine to induce immunity. However, a clear question is how to distribute the vaccine to obtain the maximum benefit from such prophylactic protection, especially if the time to manufacture a specific vaccine is comparable to the expected epidemic duration. Similarly, applied questions could focus on vaccination against livestock infections (such as foot-and-mouth disease) or wildlife diseases, where supplies are again limited and where nationally optimal policy make take precedent over equality of vaccine distribution.

Here we have re-iterated how the well-known and much-used formulation for the final-size of an epidemic (or the total number of expected cases) can be readily extended to deal with complex heterogeneous populations. The ability to calculate the final-size rapidly (without having to rely on direct simulation/integration) allows the optimal deployment of a fixed stockpile of vaccine to be determined with relative ease.

We initially focussed on the simplest situation, that of separate, non-interacting communities. For this situation, a general rule for the optimal deployment of vaccine could be determined: achieving the critical vaccination threshold in the largest possible populations (if there is sufficient vaccine to achieve this) whilst distributing the remainder of the vaccine to the smallest remaining populations. As such, for a very small vaccine stockpile, the optimal solution is to concentrate the vaccine exclusively on the smallest populations. This is comparable to the complex results found in other studies (Forster and Gilligan, 2007; Rowthorn et al., 2009; Klepac et al., 2011) although the underlying mechanism and quantities optimised are difference. All three of these papers examine endemic and on-going control, rather than epidemic infections and prophylactic vaccination. Both Forster and Gilligan (2007) and Rowthorn et al. (2009) consider the SIS model, and consider treatment of infected individuals, in brief they find that it is optimal to treat the population that currently has the fewest infected individuals. Klepac et al. (2011) focusses on endemic SIR infections in two patches with complex vaccination costs, and observes switching as the global budget for vaccine increases which is highly reminiscent of our findings.

The findings here however contrast to the well known results on vaccination in households (Becker and Starczak, 1997; Ball et al., 2004), which can be considered as very small communities. These household results acknowledge the stochastic nature of transmission process, which is necessary for small population sizes. For households the optimised schedule is that the vaccine should be sequentially targetted at the households with the largest number of remaining unvaccinated (susceptible) individuals within them. These differences are most likely attributable to the nature of transmission in the two models – as the household models will hold even for large populations when the impact of stochasticity is minimal. In household models it is generally assumed that transmission is density-dependent (despite the evidence of more complex behaviour (Cauchemez et al., 2009; Fraser et al., 2011)) such that the expected number of secondary cases produced by an initial infected individual increases with household size, whereas population-level models generally assume frequency-dependent transmission. It is still an open problem to ascertain the optimal deployment of vaccination in households when transmission deviates from this density-dependent ideal. There is therefore much scope for future work in understanding how to deal more generally with relatively small population sizes, when the impact of stochasticity and the distribution of infectious periods play a significant role. However, it should be noted that even from population sizes of only a thousand individuals, the deterministic calculation of the final epidemic size produces a close approximation to the stochastic value as long as the epidemic takes-off and we are not too close to the threshold value of *R*_0_ = 1 (Bailey, 1953; Ludwig, 1975).

Although the simple targetting scheme for prophylactic vaccination is optimal (in terms of reducing the expected number of cases), it would be considered by most as highly unacceptable. The idea that certain communities might receive vaccination whilst others are left unprotected would clearly be unacceptable to the general population (especially those that did not receive the vaccine). This inconsistency between optimal and acceptable policy could be problematic; fortunately the difference between what is acceptable and what is optimal reduces as greater realism is included. The initial investigation assumed that the two populations behaved independently, however as coupling is introduced (so that infection can pass between the populations) then there is an increased region of parameter-space where the optimal policy is to vaccinate equal proportions of the two populations. We also considered adding heterogeneity in terms of risk groups within each population; again this leads to a more uniform vaccination policy being optimal for a substantial range of vaccine availability and even when the optimal policy is not uniform there is little heterogeneity in the targetting of the at-risk group.

The way the models have been formulated means that they are caricatures of real-world vaccination policies. Although we believe the general finding are robust, a number of changes would be necessary before such models could become practical public-health tools. Throughout we have assumed that the vaccine offers complete protection against infection; if this is not true but instead vaccinated individuals have reduced susceptibility, transmissibility or consequences of infection, (a so-called leaky-vaccine (Ball et al., 2004; Arino et al., 2004)) then we need to adopt a structured population approach (Appendix A). Additionally, we have assumed throughout that it is possible to vaccinate, immunise and therefore protect any chosen fraction of the population; in practise only a proportion of those vaccinated will be successfully protected, there are often difficulties in achieving vaccination targets (White et al., 1992; Poland, 2010) and there are groups who will refuse to be vaccinated. Such effects can be incorporated into the optimisation process, by placing very high levels of immunisation out of reach – but the precise bounds would be pathogen and scenario dependent. Finally, we have assumed throughout a very simplified time-line in which a fixed stockpile of vaccine is available to be used prophylactically before an epidemic, and then the epidemic ensues in ‘free-fall’ without any additional control measures. Whilst the first of these assumptions may be valid for some outbreaks – many countries hold stockpiles of pre-pandemic vaccinate against H5N1 influenza – it is likely that both additional vaccine and additional control measures will be deployed in the face of any large-scale outbreak (Ferguson et al., 2006; Germann et al., 2006; Halloran et al., 2008; Keeling and White, 2011).

Finally, we consider what practical conclusions can be drawn from this study. The first is that vaccination programmes that are both equitable and hence publicly acceptable are likely to be sub-optimal. However, if we are dealing with a livestock or wildlife infection the optimal, highly heterogeneous solution may be more practically desirable. Secondly, the addition of extra structure that moves models away from the ideal of isolated homogeneous populations, general leads to an optimal strategy where vaccination effort is more spatially uniform. Finally, although there are a range of inherent simplifying assumptions made within the framework, we believe this methodology provides a robust and adaptable tool in which to consider optimal prophylactic vaccination.

## Figures and Tables

**Figure:**
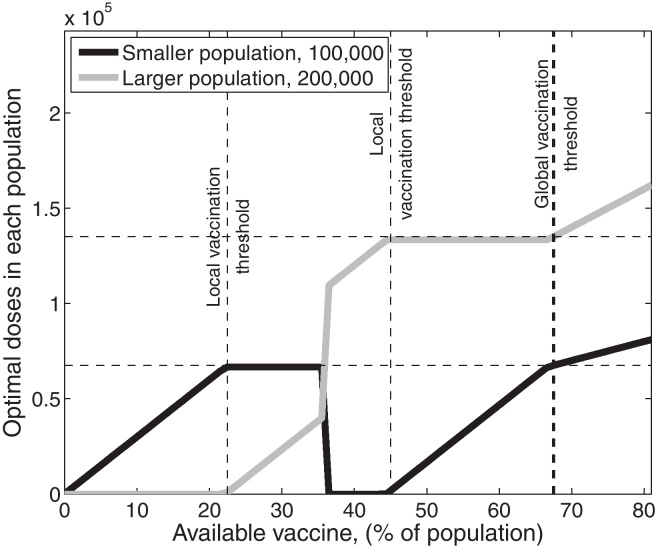


## Appendix A Final size formula for ‘leaky’ vaccines

Consider the situation where vaccination does not completely protect against infection, but instead lowers susceptibility and subsequent transmission if infected. In the simple case of a single population, the number of cases in the unvaccinated population (*Z*_∞_) and vaccinated population (*V*_∞_) are given by:

$$\begin{array}{c} Z_{\infty }=S_{0}\left[1-\exp\left(-\frac{R_{0}}{N}Z_{\infty }-\tau \frac{R_{0}}{N}V_{\infty }\right)\right]\\ V_{\infty }=V_{0}\left[1-\exp\left(-\sigma \frac{R_{0}}{N}Z_{\infty }-\tau \sigma \frac{R_{0}}{N}V_{\infty }\right)\right]=V_{0}\left[1-{\left(1-\frac{Z_{\infty }}{S_{0}}\right)}^{\sigma }\right]\\ \Rightarrow Z_{\infty }=S_{0}\left[1-\exp\left(-\frac{R_{0}}{N}Z_{\infty }-\tau \frac{R_{0}}{N}V_{0}\left\{1-{\left(1-\frac{Z_{\infty }}{S_{0}}\right)}^{\sigma }\right\}\right)\right] \end{array}$$

where *S*_0_ = *N* − *V* and *V*_0_ = *V* are the number of susceptible and vaccinated individuals in the population at the start of the epidemic. The parameters *σ* and *τ* are the relative susceptibility and transmissibility of a vaccinated individual compared to an unvaccinated individual. These equations can now be solved iteratively as before and expanded to multiple populations.

Optimal distribution of vaccine; parameters are as in Fig. 1a expect that vaccinated individuals are assumed to have half the susceptibility and half the transmissibility of unvaccinated individuals (*σ* = *τ* = 0.5).

The above figure shows that the impact of leaky vaccination on the optimal allocation of vaccination is minimal; comparing Fig. 1a in the main-paper with the above figure, it is clear that making the vaccine leaky simply acts to re-scale the points at which changes in the optimal allocation occur.

The more involved situation where a proportion *p* of vaccinated individuals are completely protected, whilst the remainder have lowered susceptibility and transmissibility can also be dealt with in a similar manner, setting *S*_0_ = *N* − *V* and *V*_0_ = (1 − *p*)*V* whilst the remaining *pV* are fully protected.
